# Understanding the potential impact of continued seed treatment use for resistance management in Cry51Aa2.834_16 *Bt* cotton against *Frankliniella fusca*

**DOI:** 10.1371/journal.pone.0239910

**Published:** 2020-10-01

**Authors:** Anders S. Huseth, Damon A. D’Ambrosio, George G. Kennedy

**Affiliations:** Department of Entomology and Plant Pathology, North Carolina State University, Raleigh, North Carolina, United States of America; Chinese Academy of Agricultural Sciences Institute of Plant Protection, CHINA

## Abstract

Transgenic cotton expressing Cry51Aa2.834_16 *Bt* toxin (hereafter referred to as MON 88702) has the potential to be an important tool for pest management due to its unique activity against tobacco thrips, *Frankliniella fusca*. Unlike other *Bt* toxins targeting lepidopteran cotton pests, MON 88702 does not cause direct mortality but has an antixenotic effect that suppresses *F*. *fusca* oviposition. Previous work has shown neonicotinoid seed treated (NST) crops have similar behavioral effects on thrips. This study used non-choice and common garden experiments to examine how the presence of MON 88702 cotton and soybean (another *F*. *fusca* host) with and without NSTs might alter *F*. *fusca* infestation distributions. In a no-choice environment, significant larval establishment differences were observed, with untreated soybean plants becoming most heavily infested. In choice experiments, plants expressing MON 88702 or were neonicotinoid treated had significantly lower larval establishment. Larval density decreased as dispersal distance increased, suggesting reproductive decisions were negatively related to distance from the release point. Understanding how *F*. *fusca* responds to MON 88702 in an environment where adults can choose among multiple host plants will provide valuable context for projections regarding design of MON 88702 resistance refuges. Reduced larval establishment on NST cotton and soybean suggests that area-wide use of NSTs could reduce the number of susceptible *F*. *fusca* generated in unstructured crop refuges for MON 88702. These results also suggest that although the presence of NST MON 88702 could suppress reproduction and resistance selection, over time this benefit could erode resulting in increased larval establishment on NST cotton and soybean due to increased frequency of neonicotinoid resistant *F*. *fusca* populations.

## Introduction

Genetically engineered cotton varieties that express *Bacillus thuringiensis* (*Bt*) toxins are used to manage lepidopteran pests throughout the world. Over the past two decades, *Bt* toxins have become a cornerstone of integrated pest management in cotton and have, in turn, driven a reduction in the prevalence of target pests and the use of insecticides to manage them, while increasing beneficial invertebrate abundance in the crop [1–4]. Despite the success of *Bt* cotton against primary lepidopteran pests, ongoing secondary pest infestations continue to require substantial insecticide inputs, often reducing these important benefits [5–7]. To address part of this secondary pest gap, Bayer Crop Science has developed a novel Cry51Aa2.834_16 *Bt* toxin expressed in cotton to target both hemipteran and thysanopteran pests [8]. In the U.S. Cotton Belt, the Cry51Aa2.834_16 *Bt* toxin (hereafter referred to as MON 88702) will be the first commercially available *Bt* toxin to control tarnished plant bugs (*Lygus lineolaris* Palisot de Beauvois; *L*. *hesperus* Knight) and thrips (*Frankliniella fusca* Hinds; *F*. *occidentalis* Pergande) [9].

Although MON 88702 cotton has documented efficacy against hemipteran and thysanopteran pests, both immature and adult stages feed on the cotton plant, which creates several opportunities for resistance selection during the pest life cycle. Moreover, because both insect groups have multiple generations in the cotton crop throughout the season [10, 11], new questions arise about the long-term strategy to mitigate resistance development after commercial deployment of MON 88702. Opportunities for selection during multiple life stages over time is a characteristic that further complicates effective resistance management strategies and will require a more refined understanding of these pests’ interaction with MON 88702 cotton and associated refuge habitats in the landscape.

In the eastern U.S., tobacco thrips (*Frankliniella fusca*) are an important early season pest of seedling cotton that causes direct damage to expanding leaf tissue, loss of apical dominance, reduced root growth, and, in cases of severe infestation, plant death [12]. To minimize seedling damage, cotton growers use a combination of at-plant neonicotinoid applications (e.g. seed treatments, in-furrow sprays) and foliar acephate applications to limit *F*. *fusca* infestations and injury [12]. In the future, MON 88702 could be an effective replacement for these at-plant and foliar insecticide applications [9]. However, the mechanism of MON 88702 activity creates added challenges to resistance management in the field. Specifically, recent studies have shown that MON 88702 acts by a non-lethal antixenosis leading to suppressed oviposition that results in fewer *F*. *fusca* larvae developing on MON 88702 cotton [13–16]. Similar non-lethal suppression of oviposition by adult *F*. *fusca* in conjunction with acute larval toxicity have been documented for neonicotinoid seed treated (NST) cotton [17–19]. The importance of non-lethal behavioral effects of both these toxins on adult *F*. *fusca* led to a specific question: how will these antixenotic effects affect *F*. *fusca* larval establishment and potential for resistance selection when adults are exposed to combinations of treated (i.e., MON 88702 cotton and NST crops) and untreated host plants that are more representative of many cotton agroecosystems?

The overarching goal of the study was to characterize *F*. *fusca* larval infestations developing on MON 88702 cotton, MON 88702 + NST, NST cotton and soybean, and untreated cotton and soybean when adult *F*. *fusca* were allowed to choose among them in a common garden experimental design that allowed insects to choose among differing host types. We hypothesized that treated hosts (i.e., MON 88702 cotton, NST cotton, and NST soybean) would have reduced larval establishment when adult female *F*. *fusca* were provided alternative untreated refuge host plants in the same common garden. We also measured larval establishment on individual host plant treatments under no-choice conditions. Outcomes of this study highlight the importance of understanding behavioral avoidance of insecticidal toxins by highly mobile pests that directly feed on crops during the adult stage. Our results provide preliminary evidence that strategic decisions to reduce NST use in key alternate host crops could benefit the long-term durability of MON 88702 in cotton production systems.

## Materials and methods

### Plants and insecticidal treatments

Treatments were partitioned by plant type (soybean, non-*Bt* cotton, MON88702) and NST insecticide (NST, no NST) combinations designed to document the oviposition choices of adult *F*. *fusca* female thrips under choice and no-choice conditions (Table 1). Soybean was included because it is widely grown throughout the U.S. Cotton Belt and is an important host of *F*. *fusca*. Like cotton, soybean is also commonly grown using NST seed [20–22]. In both no-choice and choice experiments, we used a standardized infestation level of 3 adult female *F*. *fusca* per seedling; this density is commonly observed on seedling cotton under moderate to high thrips pressure in the eastern U.S. Cotton Belt.

**Table 1:** Details on plant types and insecticidal evaluated in choice and no-choice experiments.

Plant type	Cultivar	*Bt* toxin	Seed treatment
Cotton	MON 88702	Cry51Aa2.834_16	0.375 mg imidacloprid seed^-1^
Cotton	MON 88702	Cry51Aa2.834_16	-
Cotton	DP 393	-	-
Soybean	AG 4831	-	0.18 mg imidacloprid seed^-1^
Soybean	AG 4831	-	-

We used a near-isoline (Deltapine^®^ 393) cotton variety to enable the most direct comparison possible between *Bt* and non-*Bt* cotton cultivars. DP 393 (hereafter referred to as non-*Bt* cotton) was provided by Bayer Crop Science for the purposes of comparison to MON 88702 in this study. NST-treated MON 88702 was treated with 0.375 mg imidacloprid per seed (Gaucho^®^ 600FS, 600 g imidacloprid L^-1^, Bayer Crop Science, Research Triangle Park, NC USA). NST-treated non-*Bt* cotton was not included in this study. Although NST-treated cotton varieties that do not express the MON 88702 trait will be common during the initial deployment, our overarching question involved the relative value of untreated, non-*Bt* cotton and soybean as a refuge. To understand the relative importance of NST-treated and untreated soybean as a *F*. *fusca* host, we selected a common soybean cultivar in North Carolina, Asgrow 4831 (Bayer Crop Science, St. Louis, MO USA). Neonicotinoid-treated AG 4831 soybean seeds received 0.18 mg imidacloprid per seed (Acceleron^®^ IX-409, 600 g imidacloprid L^-1^, Bayer Crop Science, St. Louis, MO USA). The amount of imidacloprid per seed for both cotton and soybean was based on two common insecticide rates for commercial crop production in our region.

### No-choice experiment

To document baseline reproductive suitability of host plants and treatments, we estimated larval infestations per seedling that developed on each plant type and insecticidal toxin combination in a greenhouse experiment following release of adult female *F*. *fusca* under no-choice conditions (Table 1). To do this, 25 ten-day-old seedlings of each treatment were grown in individual 15.2 cm-diameter clay pots that contained commercial potting mixture (Fafard 4P Mix, Sungro Horticulture, Agawam, MA, USA). Pots were completely randomized across two greenhouse benches. Each pot was equipped with a thrips-proof enclosure constructed of a modified 2 L beverage bottle equipped with 100 μm thrips-proof nylon monofilament mesh vents to allow for airflow (NMO100, Midwest Filter, St. Charles, IL, USA). Individual seedlings received water through an automated system delivering ca. 63.5 mL of water per pot over a 3-minute interval every 6 h (ca. 250 mL day^-1^). This maintained adequate soil moisture without leaching of insecticide through the pot. Greenhouse conditions were maintained at 32°C under natural lighting for ten days before infestation. Under these conditions, cotton seedlings developed to the emergence of the first true leaf, and soybeans to the emergence of the first trifoliate.

Individual seedlings were moved to a controlled environment of 23.5°C under constant light and infested with three adult female *F*. *fusca* obtained from a neonicotinoid-susceptible, laboratory-reared colony maintained on white cabbage (*Brassica oleracea* L.). To maximize reproductive potential, insects were selected ca. 2 days after becoming adults. After ten days, seedlings were harvested and washed through a series of soil sieves to collect larval thrips using the methods of Rummel and Arnold [23]. Briefly, harvested seedling samples were first washed over a 500 μm sieve to separate immature thrips from plant matter and large debris, followed by a 150 μm sieve, which collected the thrips larvae. Thrips were then rinsed from the fine sieve with 70% ethanol into 20 mL scintillation vials. *Frankliniella fusca* larvae were counted using a stereomicroscope.

### Choice experiments

All seedlings for *F*. *fusca* choice experiments were germinated and maintained in square 16-cell Styrofoam float trays designed for growing seedlings in hydroponic float systems. Trays were constructed by cutting commercial 8 x 16-cell float trays (128-cell CGP Float Tray, Carolina Greenhouses, Kinston, NC, USA) into 4 x 4-cell square blocks using a heated cutting knife. Cells were 16 cm^2^ and contained commercial potting mixture (Table 1, Fafard 4P Mix, Sungro Horticulture, Agawam, MA, USA). Each 16-cell float tray was planted with a single treatment prior to placement in a deep water-culture, raft system on a greenhouse table. Individual trays floated so that the soil passively imbibed water. Greenhouse conditions were maintained at 32°C under natural lighting for ten days before starting choice experiments. Under these conditions, cotton seedlings had developed to the emergence of the first true leaf, and soybeans to the emergence of the first trifoliate, when placed in experimental arenas.

To understand the effect of treatments on combined effects of adult *F*. *fusca* host selection and larval establishment, we conducted four temporal replicates of a choice experiment. Each temporal replicate included eight independent large cage replications that contained two seedling trays from each treatment (Table 1). Choice cages were 160 x 90 cm (width x height) open-bottomed cylinders constructed of 100 μm thrips-proof nylon monofilament mesh (NMO100, Midwest Filter, St. Charles, IL, USA). A single, vertical zipper closure was sewn along one edge to allow access to the cage interior. Each cage had six equally spaced loops sewn along the circumference, which were used to hang each screen cage on a circular PVC frame. Cage bottoms were sealed using adhesive tape to prevent insect escape. The center point of each cage interior was determined and two concentric rings (60 & 120 cm diameter) were marked. Using this design, trays were located at a radius of either 30 or 60 cm from the central *F*. *fusca* release point. Along each ring circumference, five locations were marked at equal 72° intervals. The marks between the interior and exterior rings were offset by 36°, allowing for a clear line-of-sight from each of the ten seedling tray locations to the central insect release point of the cage.

Seedling trays of each treatment were randomly assigned to locations on each concentric ring, ensuring one replicate of each treatment was placed at 30 and 60 cm from the central thrips release point (one treatment by distance replicate in each cage). Neonicotinoid susceptible, adult female *F*. *fusca* were aspirated into 1.5 mL Eppendorf tubes in groups of 96 individuals. Five tubes of insects were released from a Styrofoam tube rack placed at the center point of each cage (N = 480 individuals per cage or approximately 3 adults per seedling). Insects were allowed to infest plants for 10 d under controlled conditions of 23.5°C with constant, uniform, overhead lighting. In total, the eight cage replicates per temporal replicate, and four temporal replicates, generated 32 replicates for each unique treatment by concentric ring combination.

At the end of the infestation period, seedlings were destructively harvested by clipping the hypocotyl flush with the soil. The total number of seedlings in each tray was recorded and harvested seedlings were placed into 260 mL plastic jars (#128070TSPP, Mold-Rite Plastics, Plattsburgh, NY, USA) containing 150 mL water and 250 μL liquid detergent. The median seedling count per tray was 15 across all four choice experiment replications (13 first quartile, 16 third quartile). Larvae were washed as described in the no-choice experimental methods above. The total number of larvae were divided by the total number of seedlings in each individual treatment tray to calculate the average number of thrips larvae per seedling (average larvae per treatment by release distance).

### Statistical analysis

Generalized linear mixed models were used to test for differences in *F*. *fusca* larval establishment among treatments using PROC GLIMMIX in SAS Version 9.4 (SAS Institute, Cary, NC, USA). The response variable was the average count of *F*. *fusca* larvae per seedling, which was log(x+1) transformed to meet assumptions of normality. The no-choice model tested a fixed effect of treatment using one-way ANOVA. For choice experiments, the analysis tested categorical fixed effects of treatment and a continuous effect for distance, along with their interaction using a two-way ANOVA. The choice experiment mixed model included temporal replication and cage nested within temporal replication as random effects. For both the choice and no-choice experiments, Tukey’s Honestly Significant Difference (HSD) tests were used to compare least squares means among treatments at a significance level of α = 0.05. All summary statistics and figures were generated in R (R-Core, Version 3.4.3).

## Results

### No-choice experiment

**Fig 1:**
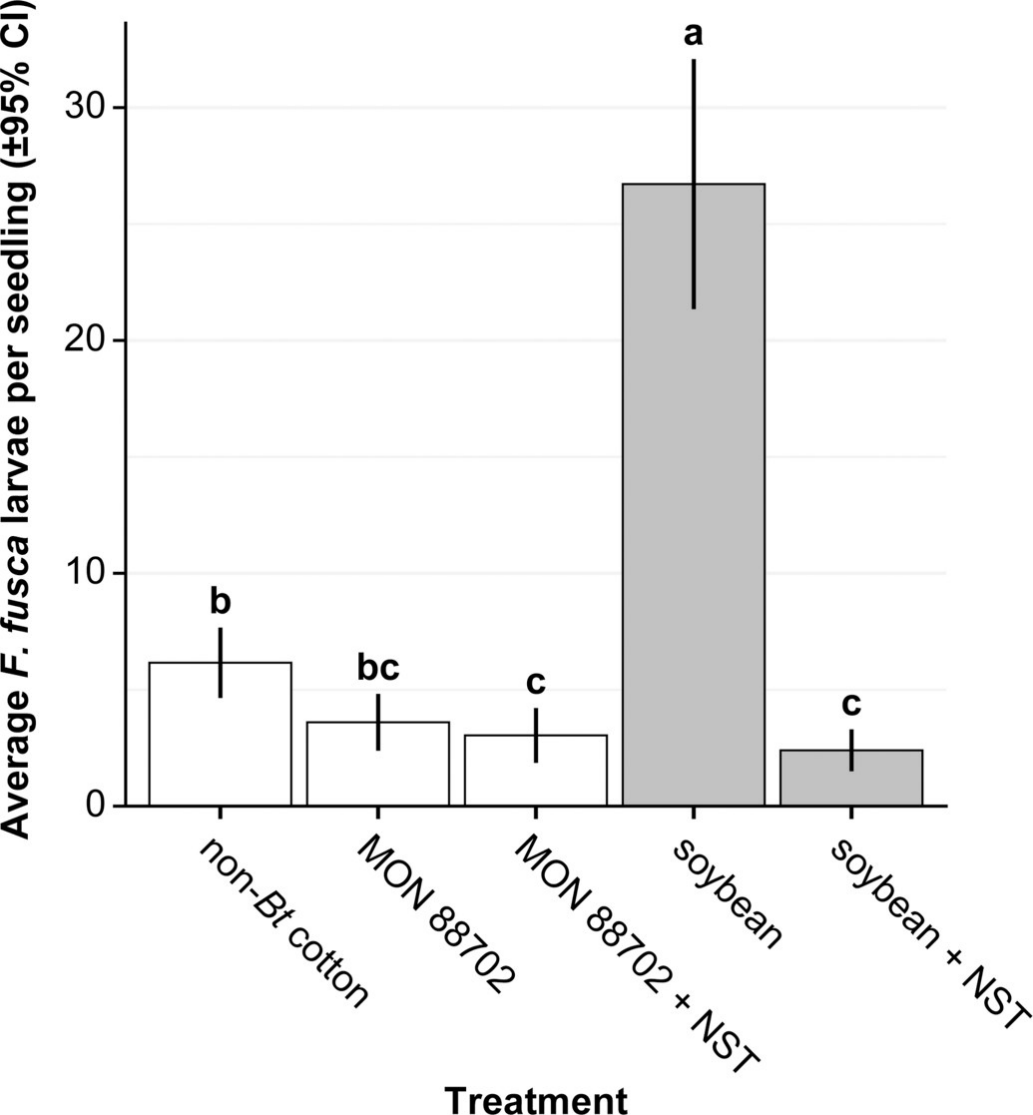
Effect of plant type and treatment on *Frankliniella fusca* larval establishment per seedling under no-choice conditions. Each treatment group consisted of 25 individual seedling replicates. Treatments with different letters above bars differed significantly from each other (Tukey’s HSD tests, *P* ≤ 0.05).

*Frankliniella fusca* larval establishment was significantly different among host plant treatments (*F*_4,120_ = 39.2; *P* < 0.001). Treatment means separation tests indicated *F*. *fusca* infestations were highest on untreated soybean (Fig 1). In this experiment, 10.1-fold more *F*. *fusca* larvae were recovered from untreated than NST-soybean (Table 2). Moreover, *F*. *fusca* larval establishment between untreated soybean and MON 88702 with or without NST were significantly different (Fig 1 and Table 2). In contrast, a 0.2-fold difference in larval establishment was observed between untreated and neonicotinoid treated MON 88702 cotton (Table 2). These differences highlight clear effects of insecticidal toxins on thrips larval establishment. Comparison of untreated MON 88702 and NST soybean were not statistically different and only resulted in a small difference in average larval establishment (Fig 1 and Table 2). NST soybean, NST MON 88702 and untreated MON 88702 resulted in the largest suppression of *F*. *fusca* larval establishment relative to untreated soybean (Fig 1), under no-choice conditions.

**Table 2:** Pairwise fold differences in *F*. *fusca* larval counts among treatments in no-choice and choice experiments.

Group One	Group Two	Choice fold difference^a^	Choice fold difference rank^b^	No-choice fold difference	No-choice fold difference rank
Soybean	MON 88702 + NST	5.9	1	7.8	2
DP393	MON 88702 + NST	4.6	2	1	6
Soybean	soybean + NST	2.9	3	10.1	1
MON 88702	MON 88702 + NST	2.5	4	0.2	8
DP393	Soybean + NST	2.1	5	1.6	5
MON 88702	Soybean + NST	1	6	0.5	8
Soybean	MON 88702	0.9	7	6.4	3
Soybean + NST	MON 88702 + NST	0.8	8	-0.2	10
DP393	MON 88702	0.6	9	0.7	7
Soybean	DP393	0.2	10	3.3	4

^a^Fold Difference = (group_1_—group_2_)/group_2_.

^b^Pairwise differences have been ordered from largest to smallest differences in choice experiments.

### Choice experiments

**Fig 2:**
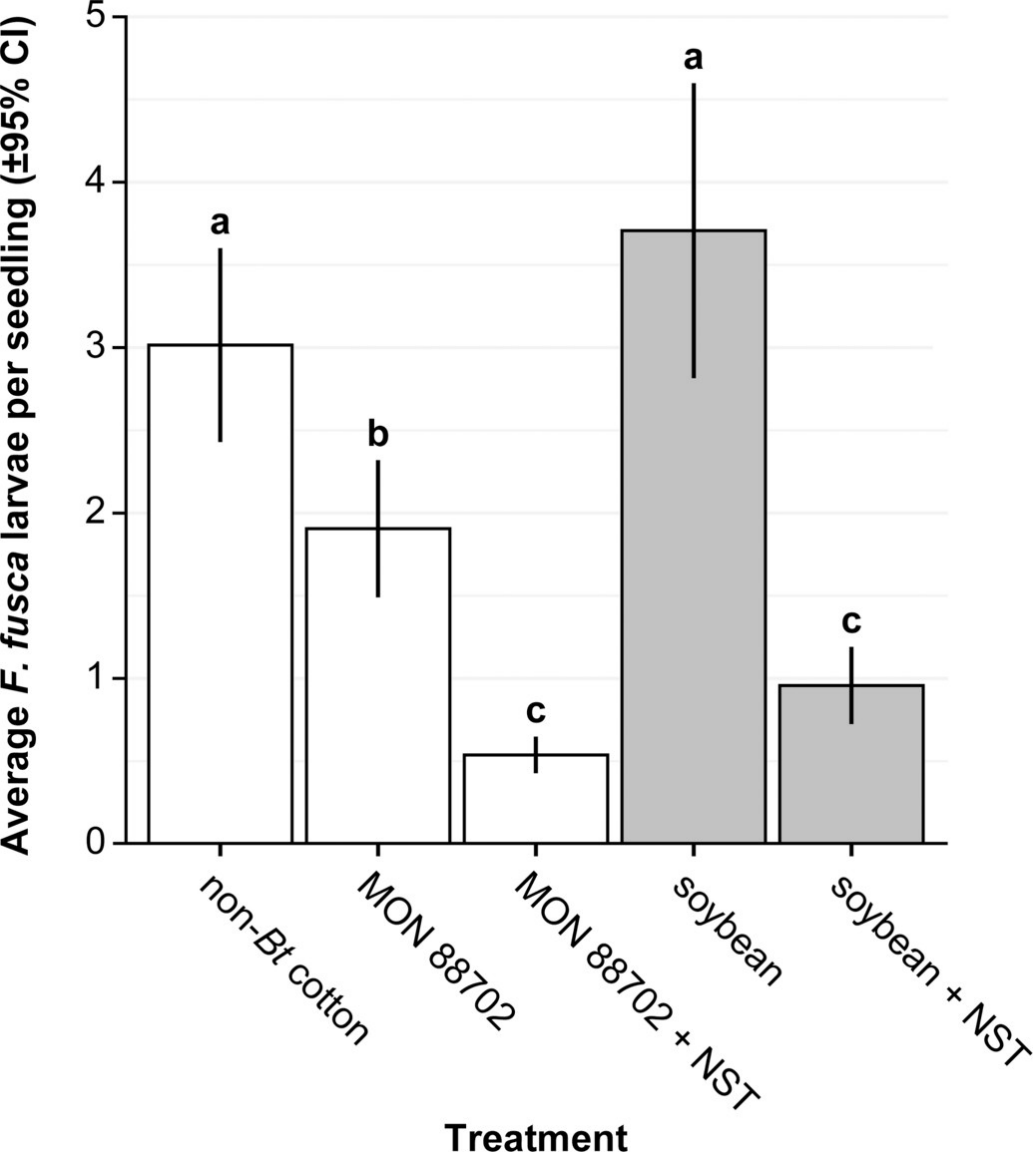
Average *Frankliniella fusca* larvae per treatment across all four temporal replicates of the common-garden experiment in which adults were allowed to oviposit freely across treatments. Treatments with different letters above bars differed significantly from each other (Tukey’s HSD tests, *P* ≤ 0.05).

In this study, we documented a strong effect of plant treatment on larval establishment in common garden cages (F_4,229_ = 22.49; *P* < 0.001). Treatments that did not include MON 88702 or a neonicotinoid had the greatest average larval establishment (Fig 2). Select treatment comparisons revealed considerable differences in average larval establishment between NST-treated plants and their untreated comparison (Table 2). Similar levels of larval establishment were observed between soybean and non-*Bt* cotton in the absence of insecticidal components. The same pattern was observed between NST-treated MON 88702 cotton and NST-treated soybean, with infestations on non-treated MON 88702 cotton being intermediate (Fig 2). Fold-change values highlight the large differences among main effect treatments (Table 2).

**Fig 3:**
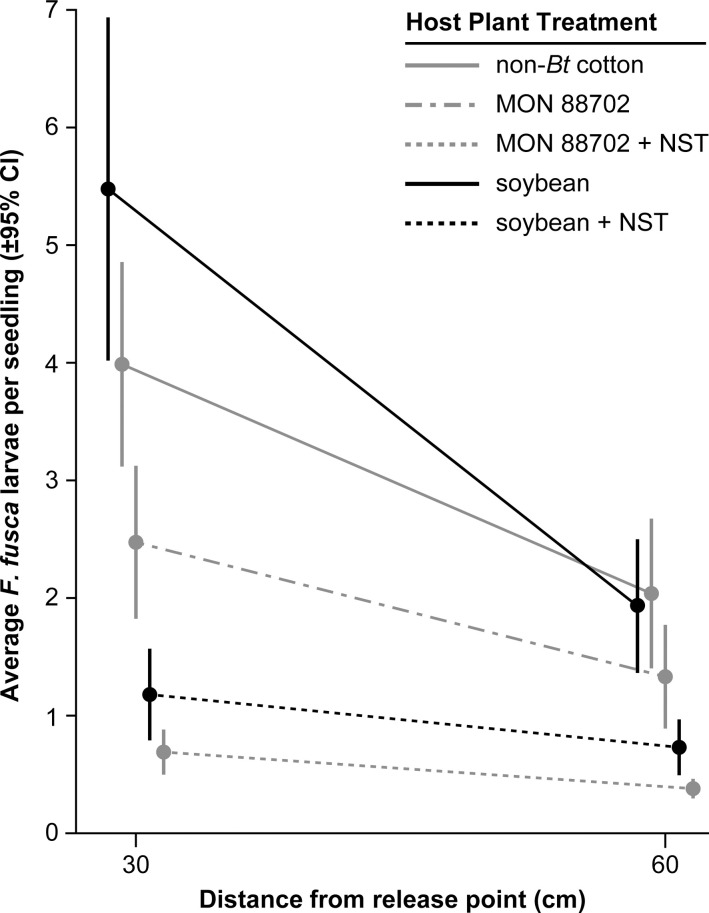
Average larval *Frankliniella fusca* per seedling from each treatment by distance from release point. Distance from release point is a continuous variable. Means and error bars have been jittered to improve the visualization of treatment comparison within and between concentric rings.

Using our ring design, we determined that host plant proximity to the initial release point affected adult *F*. *fusca* reproductive host decisions and subsequent larval establishment. The negative effect of distance from the central release point was highly significant (*F*_1,229_ = 41.6; *P* < 0.001), as was the treatment by distance interaction (*F*_4,229_ = 5.6; *P* < 0.001). Host plant selection was more discriminating close to the central release point, as indicated by greater differences in larval numbers among treatments at 30 than at 60 cm from the release point (Fig 3). Interestingly, the effect of distance on *F*. *fusca* larval establishment was minimal between two NST-containing treatments, which caused this significant interaction (Fig 3).

## Discussion

Both MON 88702 cotton and neonicotinoid seed treatments have strong antixenotic effects on adult *F*. *fusca* resulting in large reductions in oviposition and larval establishment on cotton, even in the presence of alternative host plants. While MON 88702 alters feeding behavior and reduces oviposition of adults, it has minimal impacts on adult and larval survival [13–16]. Both imidacloprid and thiamethoxam applied as seed treatments also have minimal impact on survival of adult *F*. *fusca* and dramatically reduce oviposition on treated cotton seedlings; however, they also cause moderate to high levels of larval mortality [17–19]. Because NST’s are used on ca. 87% of the soybean and 90% of cotton acreage [24–26] in the region and MON 88702 has the potential to replace a significant portion of this use in cotton, this study was undertaken to examine how the presence of MON 88702 cotton and soybean with and without NSTs in a landscape might alter *F*. *fusca* infestation distributions.

Our results show an effect of MON 88702 combined with imidacloprid seed treatment on the larval populations developing on seedlings in both choice and non-choice experiments, but the number of significant differences among treatments was greater when adults were allowed to select plants on which to oviposit. In the no-choice experiment, larval infestations on soybean (no NST) were greater than those on non-*Bt* cotton (no NST) (Table 2). Differences between non-treated soybean and MON 88702 with and without NST and the soybean + NST treatments were of even greater magnitude but differences among these latter three treatments were small and not significant (Fig 1 and Table 2).

In our common garden experiment, thrips larval density decreased with distance from the adult release point. The magnitude of differences among the soybean, non-*Bt* cotton, and MON 88702 treatments without NST were greater close to the adult release point. In contrast, larval densities on MON 88702 and soybean treatments with NST were consistently low and similar at both distances (Fig 3), likely reflecting both the strong antixenotic effects of imidacloprid seed treatments on adult *F*. *fusca* and effects on larval mortality [17–19]. Larval infestations on soybean and non-*Bt* cotton both in the absence of NST, did not differ significantly, and the infestation on soybean in the absence of NST was only 0.9-fold greater than that on MON 88702 without NST. Although larval infestations on both MON 88702 + NST and soybean + NST were both lower than the soybean and MON 88702 treatments in the absence of NST, the magnitude of the difference between these pairwise comparisons were similar (Table 2). Thus, NST treatments reduced the size of the larval infestation that developed on both MON 88702 and soybean seedlings but not the magnitude of the relative difference in infestation size between MON 88702 cotton and soybean (Table 2).

In cotton production landscapes, fields of both NST-treated MON 88702 cotton and NST-treated soybean will likely co-occur following the commercialization of MON 88702. Other possible scenarios include: presence of fields planted to NST-treated MON 88702 and non-treated soybean, a choice in our experiment that resulted in a larval infestation that was 5.9 fold greater on the non-treated soybean than on the NST-treated MON 88702 seedlings; and presence of NST-treated soybean and non-treated MON 88702, which in our choice experiment resulted in larval infestations on NST-treated soybean that were 1.0-fold lower than on MON 88702 seedlings (Table 2). These differences suggest that the pest’s interaction with different combinations of crops and insecticidal treatments may drive meaningful variation in the development of *F*. *fusca* populations across the agricultural landscape that have the potential to influence emergence of resistance to MON 88702. However, the effects we report need to be validated at the scale of commercial fields nested within a mosaic of other crops in the cotton production system.

### Potential implications for MON 88702 resistance management

To address resistance development, the MON 88702 resistance management plan will likely depend on natural (unstructured) refuge given the history of refuge design for GE cotton [27]. In practice, unstructured refuges are non-toxic habitat patches in the surrounding landscape that generate susceptible individuals to mate with resistant individuals that have developed in the *Bt* crop [28–30]. Abundant alternate crop or natural host plants in the landscape are often a predictable source of susceptible pest individuals in the southeastern U.S. [31, 32]. However, treatment of refuges with insecticides is one confounding factor that can reduce the overall viability of these host patches for pest reproduction [33]. The negative impact of widespread insecticide-treatment of alternate host crops could be amplified in intensive agricultural production systems that have limited non-crop plant hosts available. In lepidopteran systems, researchers have shown that insecticide treatment of non-*Bt* crop refuge can undermine its overall productivity, thereby reducing the efficacy of the structured refuge system as a whole in delaying resistance onset [33, 34]. In the case of MON 88702 cotton, the designated refuge would include soybean, and the effects of ongoing NST in soybean use would negatively impact refuge viability. The value of NSTs in soybean production is unclear; a series of studies has documented little or no benefit of NST use in protecting soybean from yield losses [22, 35]. In the absence of a yield cost, reduction in NST use on soybean would not only reduce grower inputs and potential environmental impacts of the soybean system itself but also increase the effective refuge area for MON 88702; thereby providing similar benefits in the cotton system as well.

At the landscape scale, numerous studies have demonstrated that the spatiotemporal structure of these toxic and non-toxic patches can play an important role in the rate of resistance development over time [36–38]. Although neonicotinoids and MON 88702 have very different modes of action against *F*. *fusca*, the intensity of cotton and alternate crop production in the U.S. Cotton Belt could provide insight into the potential for MON 88702 resistance development in the region. We know that selection for neonicotinoid resistance in *F*. *fusca* was driven in part by widespread NST use in both cotton and soybean, two key host plants for this pest [39, 40]. The importance of this cross-crop resistance selection between NST cotton and NST soybean was not entirely clear until the emergence of neonicotinoid resistance throughout the region [40]. In contrast to the *F*. *fusca* neonicotinoid resistance situation, the absence of thrips-active *Bt* toxins in soybean may provide an important constituent of an unstructured refuge for MON 88702 susceptible thrips. However, the widespread use of NSTs in soybean may effectively reduce the role of soybean as an abundant refuge patch and compromise the functional value of unstructured crop refuges for MON 88702 cotton.

## Supporting information

S1 Dataset NoChoice dataset. (TXT)

S2 Dataset Common garden dataset. (TXT)
